# Internationale Umfrage zur Behandlungspraktiken bei atopischer Dermatitis bei schwangeren und stillenden Frauen: Perspektiven von Ärzten

**DOI:** 10.1111/ddg.15728_g

**Published:** 2025-09-15

**Authors:** Manuel P. Pereira, Katarina Stevanovic, Emek Kocatürk, Cathrin Meesch, Ingrid van Hofman, Prema S. Vaswani, Jonathan A. Bernstein, Dayanne Bruscky, Herberto J. Chong‐Neto, Chia‐Yu Chu, Roberta Fachini Jardim Criado, Luis Felipe Ensina, Ana M. Giménez‐Arnau, Kiran Godse, Maia Gotua, Stamatios Gregoriou, Kanokvalai Kulthanan, Charlotte G. Mortz, Natasa Teovska Mitrevska, Esen Özkaya, Prajwal Pudasaini, Mara Morelo Rocha Felix, Catalina Rincón Pérez, Claudio Alberto Salvador Parisi, Gonzalo N. Ramón, Efstratios Vakirlis, Zuotao Zhao, Lisa A. Beck, Marjolein de Bruin‐Weller, Michael Cork, Norito Katoh, Thomas Werfel, Margitta Worm, Andreas Wollenberg, Torsten Zuberbier

**Affiliations:** ^1^ Institute of Allergology Charité – Universitätsmedizin Berlin Corporate Member of Freie Universität Berlin and Humboldt‐Universität zu Berlin Berlin Germany; ^2^ Fraunhofer Institute for Translational Medicine and Pharmacology ITMP Immunology and Allergology Berlin Germany; ^3^ GA2LEN Global Allergy and Asthma Excellence Network Berlin Germany; ^4^ Bahcesehir University School of Medicine Department of Dermatology Istanbul Turkey; ^5^ University of Cincinnati College of Medicine Division of Rheumatology Allergy and Immunology Partner of Bernstein Allergy Group and Bernstein Clinical Research Center; ^6^ Allergy and Immunology Research Center Federal University of Pernambuco Recife Brazil; ^7^ Division of Allergy and Immunology‐Complexo Hospital de Clínicas‐Federal University of Paraná, Brazil; ^8^ Department of Dermatology National Taiwan University Hospital and National Taiwan University College of Medicine Taipei Taiwan; ^9^ Department of Dermatology Centro Universitário FMABC Alergoskin Alergia e Dermatologia – UCARE/ADCARE Center of Excellence – GA^2^LEN Santo André Brazil; ^10^ CPAlpha Clinical Research and Allergy Center Barueri Brazil; ^11^ Department of Dermatology Hospital del Mar Research Institute Universitat Pompeu Fabra Barcelona Spain; ^12^ D Y Patil University school of medicine Navi Mumbai India; ^13^ Center of Allergy and Immunology ADCARE Center Tbilisi Georgia; ^14^ David Tvildiani Medical University Tbilisi Georgia; ^15^ 1st Department of Dermatology and Venereology Andreas Sygros Hospital Faculty of Medicine National and Kapodistrian University of Athens Athens Greece; ^16^ Department of Dermatology Faculty of Medicine Siriraj Hospital Mahidol University Siriraj ADCARE Center Bangkok Thailand; ^17^ Department of Dermatology and Allergy Center Odense University Hospital Odense Denmark; ^18^ Department of Dermatology Remedika General Hospital Skopje North Macedonia; ^19^ Department of Dermatology and Venereology İstanbul Faculty of Medicine Istanbul University Istanbul Turkey; ^20^ Civil Service Hospital ADCARE Center Government of Nepal Kathmandu Nepal; ^21^ Alergolife ADCARE Center Rio de Janeiro Brazil; ^22^ GA^2^LEN Atopic Dermatitis Center of Reference and Excellence Medical Specialty Unit, Secretaría de la Defensa Nacional Mexico City Mexico; ^23^ Pediatric and Adult Allergy Sections Italian Hospital of Buenos Aires Buenos Aires Argentina; ^24^ Instituto de Alergia e Inmunología del Sur Bahía Blanca Argentina; ^25^ 1st Department of Dermatology and Venereology Aristotle University of Thessaloniki Thessaloniki Greece; ^26^ Department of Dermatology and Venereology Peking University First Hospital Beijing Key Laboratory of Molecular Diagnosis on Dermatoses National Clinical Research Center for Skin and Immune Diseases NMPA Key Laboratory for Quality Control and Evaluation of Cosmetics Beijing China; ^27^ University of Rochester Medical Center Rochester New York USA; ^28^ Department of Dermatology and Allergology National Expertise Center for Atopic Dermatitis University Medical Center Utrecht Utrecht The Netherlands; ^29^ Sheffield Dermatology Research IICD University of Sheffield Sheffield UK; ^30^ North Campus Kyoto Prefectural University of Medicine Kyoto Japan; ^31^ Department of Dermatology and Allergy Hannover Medical School Hannover Germany; ^32^ Division of Allergy and Immunology Department of Dermatology and Allergology Charité – Universitätsmedizin Berlin Berlin Germany; ^33^ Department of Dermatology and Allergy Augsburg University Hospital Augsburg Germany; ^34^ Department of Dermatology and Allergy Ludwig Maximilian University of Munich Munich Germany; ^35^ Comprehensive Center for Inflammatory Medicine (CCIM) University Hospital Schleswig‐Holstein (UKSH) Lübeck Germany

**Keywords:** Atopische Dermatitis, Schwangerschaft, Stillen, systemische Therapie, Umfrage, Atopic dermatitis, breastfeeding, pregnancy, survey, systemic treatment

## Abstract

**Hintergrund und Ziele:**

Die systemische Behandlung von schwangeren/stillenden Patientinnen mit atopischer Dermatitis (AD) ist aufgrund begrenzter Sicherheitsdaten eine Herausforderung. Wir untersuchten die Behandlungspraktiken mit systemischen Therapeutika, einschließlich des in den Leitlinien empfohlenen Ciclosporin als erste systemische Wahl, sowie neue Therapien in dieser vulnerablen Bevölkerungsgruppe.

**Patienten und Methodik:**

Die ADCARE‐Initiative des *Global Allergy and Asthma Excellence Network* (GA^2^LEN) sammelte weltweit Daten von Ärzten, die schwangere Frauen mit AD behandeln. Die Ärzte füllten einen elektronischen Fragebogen zur Anwendung systemischer Therapeutika bei schwangeren/stillenden AD‐Patientinnen aus.

**Ergebnisse:**

103 Ärzte aus 32 Ländern, vor allem Dermatologen (n = 48) und Allergologen (n = 43), nahmen an der Studie teil. Antihistaminika waren das am häufigsten in Erwägung gezogene systemische Arzneimittel während der Schwangerschaft/Stillzeit (n = 73/81, 90,1%), wobei für das erste Trimester weniger Ärzte den Einsatz systemischer Wirkstoffe in Erwägung zogen als für spätere Phasen der Schwangerschaft. Bei akuten Schüben wurden systemische Kortikosteroide (n = 34/80, 42,5%) bevorzugt, gefolgt von Biologika und Antihistaminika (jeweils n = 15/80, 18,8%). Obwohl das in den Leitlinien empfohlene Ciclosporin gelegentlich für AD während der Schwangerschaft in Betracht gezogen wird (n = 38/81, 46,9%), wurde es von den Ärzten nur selten als bevorzugtes Medikament angesehen (n = 1/80, 1,25%).

**Schlussfolgerungen:**

Unsere Studie zeigt eine Diskrepanz zwischen den Leitlinienempfehlungen und den Verschreibungspraktiken und weist auf einen unbedeckten Bedarf an Wissen und der Anwendung der bestehenden Empfehlungen hin.

## EINLEITUNG

Die atopische Dermatitis ist eine chronisch‐rezidivierende, stark juckende, entzündliche Hauterkrankung.^1^, ^2^ Sie geht mit zahlreichen Komorbiditäten einher und beeinträchtigt die Lebensqualität der Patienten erheblich.^3^ Patienten leiden nicht nur unter dem sozialen Stigma einer sichtbaren Hauterkrankung, sondern auch unter starkem Pruritus, der zu Kratzläsionen und erheblichen Schlafstörungen führt. Der dadurch verursachte psychische Stress verstärkt wiederum Pruritus und Ekzem – ein Teufelskreis entsteht.^1^, ^4^ Die Behandlung der atopischen Dermatitis erfordert einen multimodalen Ansatz, der verschiedene Maßnahmen umfasst: Stärkung der Hautbarriere, eine topische entzündungshemmende und juckreizlindernde Therapie, antibakterielle Strategien sowie systemische Therapien. Zu Letzteren zählen moderne Biologika wie Dupilumab, Tralokinumab und Lebrikizumab, Januskinase (JAK)‐Inhibitoren wie Abrocitinib, Baricitinib und Upadacitinib sowie klassische Immunsuppressiva, darunter Ciclosporin, Methotrexat und Azathioprin. Diese Therapien tragen zur Krankheitskontrolle und Vorbeugung von Komorbiditäten bei.^1^, ^5^, ^6^

Die atopische Dermatitis ist die häufigste Hauterkrankung während der Schwangerschaft und macht bis zu 50% aller schwangerschaftsbedingten Dermatosen aus.^7^, ^8^ Veränderungen des Hormonspiegels beeinflussen das Zytokin‐Gleichgewicht und können zu einer erneuten Manifestation ekzematöser Läsionen führen, die als atopische Eruption in der Schwangerschaft bezeichnet werden.^7^ Bei etwa 50% der AD‐Patientinnen verschlimmert sich eine bereits vor der Schwangerschaft bestehende AD während der Schwangerschaft.^9^

Die Behandlungsmöglichkeiten für schwangere oder stillende AD‐Patientinnen sind aufgrund teratogener Effekte oder fehlender Sicherheitsdaten aus Schwangerschaftskohorten begrenzt.^10^ Aus Umfragen im Vereinigten Königreich geht außerdem hervor, dass über 70% der schwangeren AD‐Patientinnen die Einnahme von Medikamenten bewusst ablehnen, weil sie befürchten, dem Fötus zu schaden.^11^ Bei diesen Patientinnen wird von einer Behandlung mit Antihistaminika berichtet, obwohl der AD‐induzierte Pruritus Histamin‐unabhängig ist und Antihistaminika die Entzündung nicht wesentlich beeinflussen.^7^ Demgegenüber empfehlen sowohl die *European Task Force on Atopic Dermatitis* (ETFAD) als auch die aktuellen europäischen Leitlinien für atopische Dermatitis (EuroGuiDerm‐Leitlinie) Ciclosporin als Erstlinientherapie während der Schwangerschaft oder Stillzeit in schweren Fällen.^5^, ^6^, ^10^ Angesichts neu aufkommender Therapien wurde jedoch ein Dokument mit dem Titel „Safety of dermatologic medications in pregnancy and lactation“ (Sicherheit von dermatologischen Medikamenten in Schwangerschaft und Stillzeit) erstellt, das in regelmäßigen Abständen aktualisiert wird, um die Sicherheitsprofile neuartiger Therapeutika für schwangere und stillende Frauen zu überprüfen.^12^ Darüber hinaus werden derzeit Register geführt, in denen schwangere Patientinnen mit atopischer Dermatitis erfasst werden, die mit Dupilumab, Tralokinumab, Abrocitinib oder Ruxolitinib‐Creme behandelt werden (siehe FDA Pregnancy Exposure Registries). Daten zu Behandlungsmerkmalen und Schwangerschaftsverläufen bei Frauen mit AD wurden in Dänemark und in den Vereinigten Staaten erhoben,^13^, ^14^ während neuere bevölkerungsbasierte Studien den Verlauf der Schwangerschaft bei AD‐Patientinnen untersuchten, ohne die durchgeführten Therapien zu analysieren.^15^, ^16^ Es ist nach wie vor unklar, welche systemischen Behandlungen in klinischen Einrichtungen außerhalb Europas oder der USA bevorzugt werden. Ein Konsens über die systemische Therapie während der Schwangerschaft wurde bereits etabliert.^17^ Diese Studie zielt jedoch darauf ab, den ungedeckten Bedarf in der medizinischen Ausbildung zu erkennen, indem die Behandlungspraktiken von Ärzten, die routinemäßig AD‐Patienten behandeln, auf internationaler Ebene untersucht werden.

Das ADCARE‐Netzwerk umfasst spezialisierte Zentren zur Behandlung der atopischen Dermatitis und ist dem *Global Allergy and Asthma Excellence Network* (GA^2^LEN) angeschlossen – dem größten multidisziplinären Netzwerk für Forschung und klinische Versorgung im Bereich Allergie und Asthma. GA^2^LEN fördert internationale Forschungs‐ und Bildungsaktivitäten auf diesem Gebiet.

Diese Umfrage untersuchte die systemische Behandlungsstrategien von Ärzten, die schwangere oder stillende Frauen mit AD behandeln, und ob diese Ansätze mit den lokalen Behandlungsrichtlinien und den neuesten Erkenntnissen zur Sicherheit übereinstimmen.^5^, ^6^, ^18^, ^19^, ^20^, ^21^, ^22^ Die Ergebnisse sollen einen Impuls für die Entwicklung eines globalen Konsenses zu optimalen Behandlungsansätzen der atopischen Dermatitis bei schwangeren und stillenden Patientinnen geben.

## PATIENTEN UND METHODIK

### Studiendesign

Ärzte im ADCARE‐Netzwerk, die regelmäßig AD‐Patienten behandeln, wurden eingeladen, an einer Umfrage in englischer Sprache zu systemischer Behandlung von AD während der Schwangerschaft oder Stillzeit teilzunehmen; der verwendete Fragebogen ist in Abbildung S1 im Online‐Supplement dargestellt. Die Ärztinnen und Ärzte wurden über Zielsetzung und Ablauf der Studie informiert. Ihre Teilnahme erfolgte auf freiwilliger Basis. Es ergaben sich keine Risiken oder Nachteile für die teilnehmenden Ärzte, einschließlich der Möglichkeit einer Verweigerung der Teilnahme. Die teilnehmenden Ärzte konnten jederzeit und ohne Angabe von Gründen aus dem Projekt aussteigen. Den teilnehmenden Ärzten wurde die Möglichkeit angeboten, auch Ärzte, die nicht zum ADCARE‐Netzwerk gehören, zur Teilnahme an der Umfrage einzuladen. Da an dieser Studie keine Patienten beteiligt waren, wurde auf eine Genehmigung durch die Ethikkommission verzichtet.

### Studienoutcomes

Der Fragebogen umfasste sechs Fragen, von denen in diesem Artikel die Ergebnisse zu fünf berichtet werden: *(1)* medizinische Fachrichtung der teilnehmenden Ärzte; *(2)* Anzahl schwangerer Patientinnen mit atopischer Dermatitis, die in den vergangenen 3 Jahren behandelt wurden; *(3)* systemische Behandlungsoptionen, die in jedem Trimester der Schwangerschaft oder während der Stillzeit in Betracht gezogen werden (einschließlich systemischer Kortikosteroide zur akuten beziehungsweise Langzeitanwendung; systemischer Immunsuppressiva wie Azathioprin, Ciclosporin, Methotrexat, Mycophenolatmofetil oder andere; Biologika wie Dupilumab, Tralokinumab oder andere; Januskinase (JAK)‐Inhibitoren wie Abrocitinib, Baricitinib, Upadacitinib oder andere; sedierende und nichtsedierende Antihistaminika sowie weitere Arzneimittel; *(4)* bevorzugte systemische Therapie; *(5)* Komplikationen im Zusammenhang mit dem Schwangerschaftsverlauf (zum Beispiel Frühgeburt, vorzeitige Entbindung, Fehlbildungen, fetaler Verlust oder andere).

Topische Therapien und Phototherapie wurden im Rahmen dieser Erhebung nicht berücksichtigt.

### Datenerfassung und ‐analyse

Die Daten wurden anonymisiert in einer zentralen Datenbank vom 10. Juni 2023 bis zum 23. November 2023 erfasst. Fehlende Daten wurden mittels vollständiger Fallanalyse behandelt. Statistische Analysen wurden mit *IBM SPSS Statistics for Windows, Version 27* (Armonk, NY, USA), durchgeführt. Die Daten werden als Anzahl der Fälle/Gesamtzahl der Bewertungen (Prozentsatz der Fälle) angegeben.

## ERGEBNISSE

### Teilnehmer

**ABBILDUNG 1:**
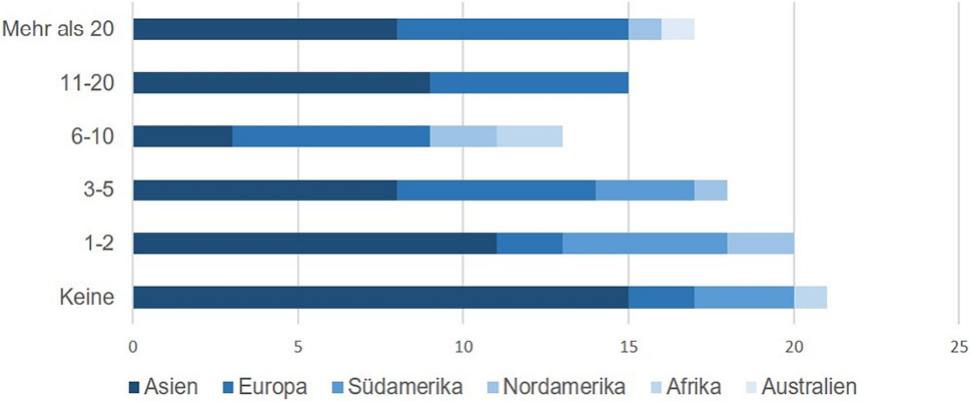
Anzahl der in den letzten drei Jahren behandelten schwangeren Patientinnen mit atopischer Dermatitis. X‐Achse: Anzahl der Befragten.

Insgesamt nahmen 103 Ärzte aus 32 Ländern auf sechs Kontinenten an der Umfrage teil. Die meisten Befragten kamen aus Asien (n = 54) und Europa (n = 28), wobei Thailand das am stärksten vertretene Land war (n = 25), gefolgt von Indien (n = 8), Brasilien (n = 8), Portugal (n = 7) und Deutschland (n = 7). Die meisten Ärzte waren entweder Dermatologen (n = 48) oder Allergologen (n = 43), mit einer breiten Verteilung hinsichtlich der Anzahl der in den letzten drei Jahren behandelten schwangeren AD‐Patientinnen (Abbildung 1). Ein Teil der Befragten (n = 81) füllte den Fragebogen vollständig aus. Die Verteilung dieser Teilnehmer nach Kontinent, Land, Fachrichtung und Erfahrung in der Behandlung schwangerer Patientinnen mit AD ist in Tabelle 1 dargestellt.

**TABELLE 1:** Verteilung der Teilnehmer nach Kontinent, Land, Fachgebiet und Erfahrung in der Behandlung schwangerer Patientinnen mit atopischer Dermatitis. Die Daten der Befragten, die den gesamten Fragebogen ausgefüllt haben, sind dargestellt.

Kontinent	Anzahl der Befragten	Land	Fachgebiet	Behandelte Patientinnen in den letzten 3 Jahren
Asien	n = 39	Thailand: n = 17, Indien: n = 5, Vietnam: n = 4, Nepal: n = 3, China: n = 2, Israel: n = 2, Japan: n = 1, Malaysia: n = 1, Philippinen: n = 1, Taiwan: n = 1, Türkei^*^: n = 1, Vereinigte Arabische Emirate: n = 1	Dermatologie: n = 17 Allergologie: n = 15 Innere Medizin: n = 1 Allgemeinmedizin: n = 6	> 20: n = 5 11–20: n = 8 6–10: n = 3 3–5: n = 6 1–2: n = 9 Keine: n = 8
Europa	n = 24	Portugal: n = 6, Deutschland: n = 5, Griechenland: n = 2, Italien: n = 2, Russland: n = 2, Türkei^*^: n = 2, Österreich: n = 1, Dänemark: n = 1, Mazedonien: n = 1, Spanien: n = 1, Schweiz: n = 1	Dermatologie: n = 15 Allergologie: n = 9	> 20: n = 6 11–20: n = 4 6–10: n = 6 3–5: n = 5 1–2: n = 2 Keine: n = 1
Südamerika	n = 10	Brasilien: n = 8, Argentinien: n = 2	Dermatologie: n = 1 Allergologie: n = 9	> 20: n = 0 11–20: n = 0 6–10: n = 0 3–5: n = 3 1–2: n = 4 Keine: n = 3
Nordamerika	n = 5	Vereinigte Staaten von Amerika: n = 3, Kanada: n = 1, Mexiko: n = 1	Dermatologie: n = 4 Allergologie: n = 1	> 20: n = 1 11–20: n = 0 6–10: n = 2 3–5: n = 1 1–2: n = 1 Keine: n = 0
Afrika	n = 2	Madagaskar: n = 1, Südafrika: n = 1	Dermatologie: n = 1 Allergologie: n = 1	> 20: n = 0 11–20: n = 0 6–10: n = 2 3–5: n = 0 1–2: n = 0 Keine: n = 0
Australien	n = 1	Australien: n = 1	Dermatologie: n = 1	> 20: n = 1 11–20: n = 0 6–10: n = 0 3–5: n = 0 1–2: n = 0 Keine: n = 0

* Die Türkei wird nach Einschätzung der Befragten entweder zu Asien oder Europa gezählt.

### Systemische Therapie

**ABBILDUNG 2:**
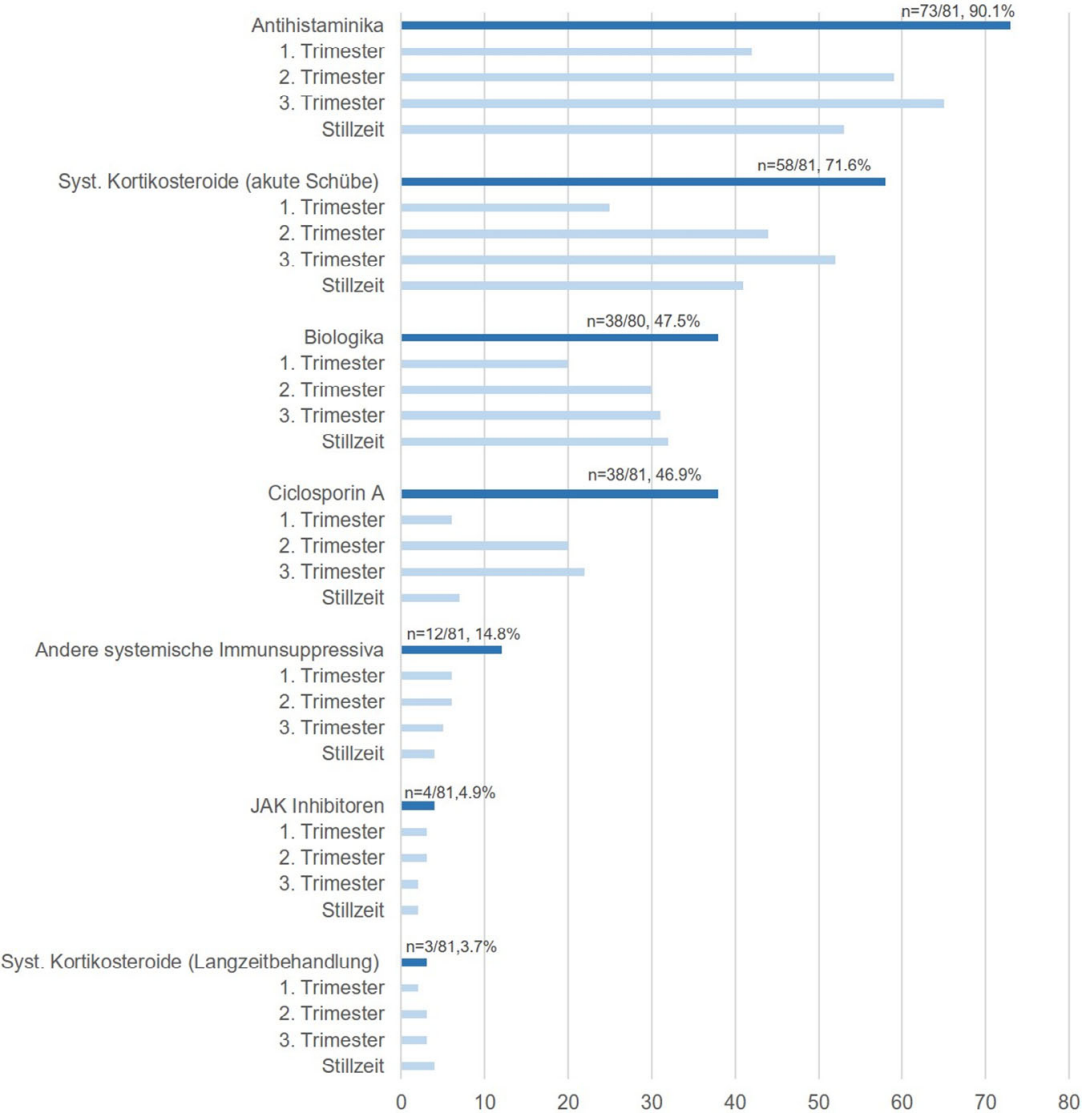
Systemische Therapien während der Schwangerschaft/Stillzeit. Dargestellt ist die Anzahl der Ärzte, die den Einsatz von Antihistaminika, systemischen Kortikosteroiden bei akuten Schüben, Biologika, systemischen Immunsuppressiva (einschließlich Ciclosporin), Januskinase (JAK)‐Inhibitoren und systemischen Kortikosteroiden als Langzeitbehandlung zur Behandlung von AD während der Schwangerschaft und Stillzeit in Erwägung ziehen. X‐Achse: Anzahl der Befragten.

Antihistaminika wurden im Allgemeinen als Therapieoption während der gesamten Schwangerschaft und Stillzeit betrachtet (n = 73/81, 90,1%), allerdings hielt eine geringere Anzahl der Befragten sie für das erste Schwangerschaftstrimester für geeignet (Abbildung 2). Meistens wurden nichtsedierende Antihistaminika (n = 56) bevorzugt, während sedierende (n = 19) Antihistaminika seltener gewählt wurden.

Auch der kurzfristige Einsatz von systemischen Kortikosteroiden bei akuten Schüben wurde von den meisten Ärzten (n = 58/81, 71,6%) für durchführbar gehalten, allerdings zogen nur 25 Ärzte deren Einsatz im ersten Trimester in Betracht (Abbildung 2).

Obwohl Biologika nicht für den Einsatz in der Schwangerschaft oder Stillzeit zugelassen sind, waren sie für eine bedeutende Anzahl von Ärzten (n = 38/80, 47,5%) eine mögliche Therapieoption, wobei Dupilumab (n = 42) das Biologikum der Wahl war, gefolgt von Tralokinumab (n = 9) und anderen Biologika (n = 2). Auch hier stuften weniger Ärzte Biologika als Behandlungsoption für das erste Trimester, als für spätere Trimester oder während der Stillzeit ein.

Eine Minderheit der Befragten betrachtete systemische Immunsuppressiva als eine Therapieoption während der Schwangerschaft oder Stillzeit (n = 19/81, 23,5%). Ciclosporin (n = 38) wurde am häufigsten in Betracht gezogen, gefolgt von Azathioprin (n = 11). Bemerkenswert ist, dass auch Immunsuppressiva oder Immunmodulatoren mit bekannter teratogener Wirkung von einigen Befragten genannt wurden: Methotrexat (n = 5), JAK‐Inhibitoren (n = 4) und Mycophenolatmofetil (n = 3).

Die langfristige Einnahme von Kortikosteroiden zur Behandlung von AD während der Schwangerschaft oder Stillzeit wurde nur selten in Betracht gezogen (n = 3/81, 3,7%), während n = 27/80 (33,8%) keine systemischen Mittel während der Schwangerschaft oder Stillzeit anwenden.

### Bevorzugte systemische Therapien

**ABBILDUNG 3:**
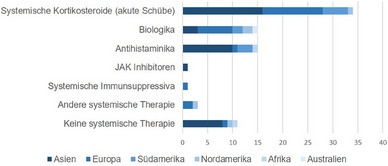
Bevorzugte systemische Behandlung der atopischen Dermatitis während der Schwangerschaft/Stillzeit. Dargestellt ist die Anzahl der Ärzte, die den Einsatz von systemischen Kortikosteroiden bei akuten Schüben, Biologika, Antihistaminika, Januskinase (JAK)‐Inhibitoren, systemischen Immunsuppressiva und anderen systemischen Therapien zur Behandlung von atopischer Dermatitis während der Schwangerschaft und Stillzeit bevorzugen. X‐Achse: Anzahl der Befragten.

Achtzig Ärzte beantworteten die Frage nach ihrer bevorzugten systemischen Behandlung von AD während der Schwangerschaft oder Stillzeit (Abbildung 3). Systemische Kortikosteroide bei akuten Schüben waren die häufigste Antwort (n = 34, 42,5%), gefolgt von Biologika (n = 15, 18,8%) und Antihistaminika (n = 15, 18,8%). Ciclosporin und Baricitinib wurden jeweils nur von einem Befragten als systemisches Arzneimittel der Wahl angegeben.

Dupilumab wurde am häufigsten als Biologikum der Wahl genannt (n = 13), während Tralokinumab von einem Befragten erwähnt wurde (n = 1) und bei einem Befragten die Daten fehlten (n = 1). Nichtsedierende Antihistaminika (n = 8) wurden im Vergleich zu sedierenden Antihistaminika (n = 3) häufiger als Antihistaminikum der Wahl angegeben.

Elf (13,8%) der Befragten gaben an, dass sie es bevorzugen, während der Schwangerschaft oder Stillzeit keine systemischen Mittel zur Behandlung von AD einzusetzen.

### Komplikationen

Nur sechs Befragte gaben an, im Zusammenhang mit der Anwendung systemischer Therapien Komplikationen beim Schwangerschaftsverlauf erlebt zu haben. Zwei Ärzte berichteten von Frühgeburten und vorzeitigen Entbindungen. Fehlbildungen, fetaler Verlust sowie andere nicht weiter spezifizierte Komplikationen wurden jeweils einmal gemeldet.

## DISKUSSION

Die Behandlung von AD‐Patientinnen während der Schwangerschaft und Stillzeit ist nach wie vor eine Herausforderung, insbesondere wenn systemische Medikamente erforderlich sind. Unsere internationale Studie zeigte weltweite Behandlungstrends durch Ärzte auf. In erster Linie wurde festgestellt, dass einige Ärzte bei der Verabreichung von systemischen Medikamenten im ersten Trimester zurückhaltend sind, da die fetale Entwicklung in dieser Phase der Schwangerschaft besonders kritisch ist. Nichtsedierende Antihistaminika sind das Medikament, das von Ärzten am häufigsten als sicher für die Verwendung während der Schwangerschaft oder Stillzeit angesehen wird, und ihre verbreitete Anwendung spiegelt die überwältigenden Sicherheitsdaten wider, die für diese Medikamentenklasse vorliegen.^23^ Antihistaminika der älteren Generation haben jedoch eine unbekannte Wirkung auf den Fötus und sollten nicht verordnet werden.^24^ Darüber hinaus zeigen Antihistaminika nur eine geringe entzündungshemmende und Pruritus‐stillende Wirkung, da nichthistaminerge Nervenfasern für die Weiterleitung des atopischen Pruritus verantwortlich sind,^25^, ^26^, ^27^ so dass ihr Nutzen in der Behandlung der AD sowohl als Monotherapie als auch als Zusatztherapie begrenzt ist.^28^ Dementsprechend werden Antihistaminika von der ETFAD‐ und EuroGuiDerm‐Leitliniengruppen nicht zur Behandlung der AD empfohlen.^5^, ^6^, ^17^

Der Einsatz von systemischen Kortikosteroiden zur Behandlung akuter Schübe wird von den meisten Ärzten befürwortet. Es gibt jedoch Bedenken hinsichtlich einer Unterdrückung der Hypothalamus‐Hypophysen‐Nebennieren‐Achse bei Neugeborenen, die durch langfristige Kortikosteroideinnahme verursacht werden kann.^17^, ^29^

Biologika, insbesondere Dupilumab, scheinen ebenfalls große Akzeptanz zu finden, wobei ihr Einsatz in späteren Phasen der Schwangerschaft bevorzugt wird, um so kritische Phasen der fetalen Entwicklung zu vermeiden. Eine retrospektive Analyse der Exposition vor oder während der ersten 6 Wochen der Schwangerschaft bei Frauen mit AD ergab jedoch, dass während des ersten Trimesters kein signifikantes arzneimittelassoziiertes Risiko bestand.^30^ Bei der Behandlung anderer Indikationen wie Asthma ist es üblich, Biologika bei Patientinnen weiterzuführen, die vor der Schwangerschaft bereits darauf angesprochen haben.^31^ Wichtig ist, dass Biologika zur Behandlung von AD die Th2‐Entzündung blockieren, die jedoch für den Erhalt der Schwangerschaft förderlich ist. Dadurch könnten sie eine Verschiebung hin zu einer Th1‐Entzündung begünstigen. Da Th1‐Zytokine mit Komplikationen wie Präeklampsie oder Frühgeburten in Verbindung gebracht werden und dem Fötus schaden könnten, sollte bei der Wahl einer Therapie, die die Th2‐Entzündung hemmt, Vorsicht geboten sein. Dennoch deuten die derzeit verfügbaren Daten nicht darauf hin, dass Th2‐blockierende Biologika die fetalen oder mütterlichen Outcomes beeinflussen, allerdings sind qualitativ hochwertige kontrollierte Studien erforderlich, um aussagekräftigere Ergebnisse zu erhalten.^32^, ^33^, ^34^ Ein höherer Anteil von Ärzten in Europa neigt dazu, Biologika einzusetzen, verglichen mit Asien. Dies deutet auf potenziell unterschiedliche Einstellungen zur systemischen Therapie während Schwangerschaft und Stillzeit in verschiedenen Kulturen hin. Zudem könnten unterschiedliche Zugangsbedingungen zu modernen systemischen Therapien und abweichende Erstattungsrichtlinien der Krankenkassen in den jeweiligen Regionen eine Rolle spielen.

Andererseits werden die meisten systemische Immunsuppressiva und JAK‐Inhibitoren von den meisten Ärzten, die diesen Fragebogen beantwortet haben, nicht bevorzugt, da einige dieser Substanzen immer noch als sehr bedenklich im Hinblick auf potenzielle Risiken für die Mutter und den Fötus angesehen werden. Wichtig ist, dass Methotrexat,^35^ Mycophenolatmofetil,^36^ JAK‐Inhibitoren,^37^ teratogen sind und in der Schwangerschaft kontraindiziert sind, wie auch in den amerikanischen AD‐Leitlinien beschrieben.^19^ Für Ciclosporin hingegen gibt es solide Belege für seine Sicherheit während der Schwangerschaft und Stillzeit,^38^, ^39^ und es wird sowohl von der ETFAD als auch von den aktuellen europäischen Leitlinien für AD empfohlen.^5^, ^6^, ^17^ Ein Expertengremium aus Kanada veröffentlichte im Jahr 2023 evidenzbasierte Empfehlungen zur systemischen Behandlung von AD, in denen Ciclosporin als die am besten belegte Option für schwangere oder stillende Frauen, die eine systemische Therapie benötigen, angesehen wird, während Methotrexat, Mycophenolatmofetil und JAK‐Inhibitoren kontraindiziert sind.^19^, ^40^ Ciclosporin wird zudem zur Behandlung anderer Indikationen wie Nierentransplantation und chronisch‐entzündliche Darmerkrankungen während der Schwangerschaft eingesetzt.^41^, ^42^, ^43^

Bemerkenswert ist, dass ein erheblicher Anteil der befragten Ärzte angab, während der Schwangerschaft oder Stillzeit keine systemischen Medikamente zur Behandlung von AD zu verwenden. Dies unterstreicht die Herausforderung, vor der Ärzte stehen, wenn sie AD bei schwangeren Patientinnen behandeln und gleichzeitig potenzielle Risiken für Mutter und Kind berücksichtigen müssen.

Zudem wurden von einzelnen Ärzten Komplikationen wie Frühgeburtlichkeit, kongenitale Fehlbildungen und fetaler Verlust berichtet. Bei unserem Studiendesign konnte keine der gemeldeten Komplikationen auf ein bestimmtes systemisches Medikament zurückgeführt werden, so dass diese Ergebnisse mit Vorsicht zu betrachten sind. Zu den weiteren Einschränkungen der Studie zählen die relativ geringe Teilnehmerzahl sowie die begrenzte globale Abdeckung, da insbesondere nur wenige Daten aus Australien (n = 1), Afrika (n = 2) und Nordamerika (n = 5) vorliegen, wie in Tabelle 1 dargestellt.

Unsere Studie zeigt somit, dass die aktuellen Empfehlungen zur systemischen Therapie der AD während der Schwangerschaft und Stillzeit nicht immer von den behandelnden Ärzten befolgt werden. Die in unserer Umfrage beobachteten Diskrepanzen lassen sich wahrscheinlich auf unterschiedliche Erfahrungsniveaus der Befragten sowie auf regionale Unterschiede in der Behandlung schwangerer und stillender Patientinnen zurückführen. Zudem könnten sowohl Ärzte als auch Patientinnen besorgt über Nebenwirkungen und Komplikationen sein, wie beispielsweise Nierenversagen infolge einer Therapie mit Ciclosporin. Daher sollten Peer‐Education‐Initiativen, wie die ADCARE‐Schulungen,^44^, ^45^ häufiger und umfassender durchgeführt werden, um eine bessere allgemeine Versorgung dieser vulnerablen Patientengruppe mit mittelschwerer bis schwerer AD zu gewährleisten. Eine Analyse nach solchen Schulungsveranstaltungen zeigte einen Anstieg der Testergebnisse um 43% im Vergleich zum Wissensstand vor der Veranstaltung.^44^ Für eine effektive Orientierung sollten Leitlinien regionale Besonderheiten berücksichtigen, darunter den Zugang zu modernen Medikamenten und die Erstattungspolitik der Krankenversicherungen. Wir haben bereits früher die Komplexität der AD und die Vorteile der Einbeziehung verschiedener Stakeholders in die Behandlung und die Patientenaufklärung erörtert,^46^ und daher die integrierten Versorgungspfade (Integrated Care Pathways) für AD als weiteres nützliches Dokument für Patienten und Ärzte entwickelt.^1^ Mit der aktuellen Studie konnte ADCARE Erkenntnisse über bestehende Wissenslücken bei Ärzten gewinnen und Themen identifizieren, die in zukünftigen Schulungsveranstaltungen verstärkt behandelt werden sollten.

### Fazit

Unsere Studie zeigte eine Diskrepanz zwischen den Leitlinienempfehlungen und den Verschreibungspraktiken von systemischen Medikamenten zur Behandlung schwangerer und stillender Frauen mit AD. Bildungsprogramme, die sich auf die Aktualisierung der Behandlung besonderer Patientengruppen konzentrieren, sowie langfristige Register zur Erfassung von Daten zur Anwendung systemischer Medikamente während der Schwangerschaft und Stillzeit, einschließlich Wirksamkeit und Sicherheit, sind erforderlich, um zukünftige Leitlinien sowie behandelnde Ärzte fundiert zu informieren.

## FINANZIERUNG

Diese Studie wurde von Almirall finanziert. Der Sponsor unterstützte die Studie finanziell, hatte aber keinen Einfluss auf die Gestaltung des Studienprotokolls, die Datenerfassung, ‐analyse und ‐interpretation sowie auf die Entscheidung zur Veröffentlichung dieses Manuskripts. Die Autoren behielten die volle Kontrolle über alle Aspekte der Studie und die Erstellung des Manuskripts.

## DANKSAGUNG

Open access Veröffentlichung ermöglicht und organisiert durch Projekt DEAL.

## INTERESSENKONFLIKT

M.P.P. erhielt Forschungsgelder von Almirall; ist Prüfarzt für Allakos, Celldex Therapeutics, Incyte, Sanofi und Trevi Therapeutics; und erhielt Beratungsvergütungen, Vortragshonorare und/oder Reisekostenerstattungen von AbbVie, Beiersdorf, Celltrion, Eli Lilly, GA^2^LEN, Galderma, Menlo Therapeutics, Novartis, P.G. Unna Academy, Sanofi, StreamedUP und Trevi Therapeutics. E.K. war als Referent und Berater für Novartis, Menarini und Pfizer tätig. M.D.B. war als Berater, Mitglied von Advisory Boards und/oder Referent für AbbVie, Almirall, Amgen, Aslan, Eli Lilly, Galderma, Leo Pharma, Pfizer, Regeneron und Sanofi‐Genzyme tätig. N.K. erhielt Honorare als Referent/Berater für Sanofi, Maruho, AbbVie, Eli Lilly Japan, Taiho Pharmaceutical, Pfizer, Mitsubishi Tanabe Pharma, Janssen Pharma, Kyowa Kirin, Celgene Japan und Otsuka Pharmaceutical sowie forschungsinitiierte Drittmittel von Mitsubishi Tanabe Pharma, Torii Pharmaceutical, Maruho, Sun Pharma, Boehringer Ingelheim Japan, Eisai und Leo Pharma. M.W. erhielt Unterstützung für Beratertätigkeiten, Vorträge und andere wissenschaftliche Aktivitäten von ALK‐Abelló Arzneimittel GmbH, Almirall, AbbVie, Eli Lilly, Mylan Germany GmbH, Bencard Allergie GmbH, Novartis AG, Biotest AG, Sanofi‐Aventis Deutschland GmbH, HAL Allergie GmbH, DBV Technologies S.A., Aimmune Therapeutics UK Limited, Regeneron Pharmaceuticals Inc. und Stallergenes GmbH. T.W. erhielt institutionelle Forschungsgelder von Almirall, Beiersdorf, LEO Pharma und Novartis; führte Beratungen für AbbVie, Almirall, Galderma, LEO, Lilly, Novartis, Pfizer und Sanofi‐Regeneron durch; hielt Vorträge bei Fortbildungsveranstaltungen, die von AbbVie, Almirall, Galderma, LEO Pharma, Lilly, Pfizer, Sanofi und Novartis unterstützt wurden; und ist an klinischen Studien beteiligt, die mit verschiedenen Pharmaunternehmen zur Entwicklung von Therapien für atopische Dermatitis durchgeführt werden. A.W. war als Berater oder bezahlter Referent tätig oder nahm an klinischen Studien teil (mit Honoraren an die Institution) im Auftrag von AbbVie, Aileens, Almirall, Amgen, Beiersdorf, Bioderma, Bioproject, Boehringer Ingelheim, Bristol Myers Squibb, Celgene, Chugai, DKSH, Eli Lilly, Galapagos, Galderma, Glenmark, GSK, Hans Karrer, Hexal, Janssen‐Cilag, Kyowa Kirin, Leo Pharma, L'Oréal, Maruho, MedImmune, MSD, Mylan, Novartis, Pfizer, Pierre Fabre, Regeneron, Sandoz, Santen, Sanofi‐Aventis und UCB. T.Z. erhielt Vortragshonorare von Amgen, AstraZeneca, AbbVie, ALK‐Abelló, Almirall, Astellas, Bayer HealthCare, Bencard, Berlin Chemie, FAES Farma, HAL Allergie GmbH, Henkel, Kryolan, Leti, L'Oréal, Meda, Menarini, Merck Sharp & Dohme, Novartis, Nuocor, Pfizer, Sanofi, Stallergenes, Takeda, Teva, UCB und Uriach. Beratungsvergütungen aus der Industrie wurden von Abivax, Almirall, Bluprint, Celldex, Celltrion, Novartis und Sanofi erhalten. Darüber hinaus bestehen unbezahlte organisatorische Tätigkeiten: Mitglied im Komitee „Allergic Rhinitis and its Impact on Asthma“ (ARIA); Vorstandsmitglied der Deutschen Gesellschaft für Allergologie und klinische Immunologie (DGAKI); Leiter der European Centre for Allergy Research Foundation (ECARF); Präsident des Global Allergy and Asthma Excellence Network (GA^2^LEN); sowie Mitglied des Komitees für Allergiediagnostik und molekulare Allergologie der World Allergy Organization (WAO). C.Y.C. erhielt Forschungsgelder von Sanofi; ist Prüfarzt für AbbVie, Amgen, Dermira, Janssen, Eli Lilly, Novartis, Oneness Biotech, Pfizer, Regeneron Pharmaceuticals Inc., Roche und Sanofi; und erhielt Beratungsvergütungen, Vortragshonorare und/oder Reisekostenerstattungen von AbbVie, Amgen, Eli Lilly, Janssen, Novartis, Pfizer, Roche, Sanofi und Viatris. A.G.A. ist oder war kürzlich als Referent und/oder Berater tätig und/oder erhielt Forschungsgelder von Almirall, Amgen, AstraZeneca, Avène, Bluprint, Celldex, Escient Pharmaceuticals, Genentech, GSK, Harmonic Bio, Instituto Carlos III–FEDER, Jaspers, Leo Pharma, Menarini, Mitsubishi Tanabe Pharma, Novartis, Sanofi–Regeneron, Septerna, Servier, Thermo Fisher Scientific, Uriach Pharma und Noucor. L.F.E. erhielt Beratungsvergütungen von Sanofi, Vortragshonorare von Sanofi, Novartis und AbbVie und nimmt an klinischen Studien von Novartis und Amgen teil. K.K. erhielt Vortragshonorare von Pfizer, Zuellig Pharma und Sanofi. E.Ö. war in beratender Funktion für Pfizer und Sanofi tätig und erhielt Vortragshonorare von beiden Unternehmen. C.R.P. ist Prüfarzt für AbbVie und erhielt Beratungsvergütungen, Vortragshonorare und/oder Reisekostenerstattungen von AbbVie, Eli Lilly, Leo Pharma und Janssen‐Cilag. K.S., C.M., I.V.H., P.V., L.B., D.B., H.C., K.G., M.G., S.G., C.M., N.T.M., P.P., M.M.R.F., C.A.S.P., G.R. und E.V. erklären, dass keine Interessenkonflikte bestehen.

## Supporting information



Supplementary information
